# Inhibition of 6-phosphogluconate Dehydrogenase Reverses Cisplatin Resistance in Ovarian and Lung Cancer

**DOI:** 10.3389/fphar.2017.00421

**Published:** 2017-06-30

**Authors:** Wujian Zheng, Qi Feng, Jiao Liu, Yanke Guo, Lvfen Gao, Ruiman Li, Meng Xu, Guizhen Yan, Zhinan Yin, Shuai Zhang, Shuangping Liu, Changliang Shan

**Affiliations:** ^1^The First Affiliated Hospital, Biomedical Translational Research Institute, Jinan UniversityGuangzhou, China; ^2^Department of Gynecology and Obstetrics, The First Affiliated Hospital, Jinan UniversityGuangzhou, China; ^3^Department of Oncology, The First Affiliated Hospital, Jinan UniversityGuangzhou, China; ^4^Lixia District People’s HospitalJinan, China; ^5^Department of Biochemistry and Molecular Biology, Medical College of Jinan UniversityGuangzhou, China; ^6^Department of Pathology, Medical School, Dalian UniversityDalian, China

**Keywords:** 6-phosphogluconate dehydrogenase, cisplatin resistance, metabolism, oxidative pentose phosphate pathway

## Abstract

Cisplatin (DDP) is currently one of the most commonly used chemotherapeutic drugs for treating ovarian and lung cancer. However, resistance to cisplatin is common and it often leads to therapy failure. In addition, the precise mechanism of cisplatin resistance is still in its infancy. In this study, we demonstrated that the oxidative pentose phosphate pathway enzyme 6-phosphogluconate dehydrogenase (6PGD) promotes cisplatin resistance. We showed that cisplatin-resistant cancer cells (C13^∗^ and A549DDP), had higher levels of 6PGD compared to their cisplatin-sensitive counterparts (OV2008 and A549). Furthermore, ovarian and lung cancer patients with higher 6PGD levels have worse survival outcomes relative to patients with lower 6PGD expression. Interestingly, we found that the upregulation of 6PGD in cisplatin-resistant cells was due to the decreased expression of miR-206 and miR-613, which we found to target this enzyme. We further demonstrate that suppressing 6PGD using shRNA, inhibitor or miR-206/miR-613, either as single agents or in combination, could sensitize cisplatin-resistant cancer cells to cisplatin treatment and thereby improving the therapeutic efficacy of cisplatin. Taken together, our results suggest that 6PGD serves as a novel potential target to overcome cisplatin resistance.

## Introduction

Cisplatin (DDP) is currently one of the most common anticancer drugs used for treating various solid tumors including testicular, head and neck, lung and ovarian cancer (Galluzzi et al., 2012; Dasari and Tchounwou, 2014). However, the therapeutic effectiveness of cisplatin is limited due to the fact that many patients acquire resistance (Galluzzi et al., 2012; Kumar et al., 2012). There is thus an urgent to explore the molecular mechanisms that contribute to cisplatin resistance in order to find ways of bypassing these obstacles.

The Warburg effect describes a phenomenon whereby cancer cells undergo metabolism reprogramming marked by increased in aerobic glycolysis and elevated lactate production (Cairns et al., 2011; Fan et al., 2014). Increasing evidence supports the notion that deregulated cellular metabolism has a close relationship with drug resistance in cancer therapy (Liu et al., 2008; Wang et al., 2010; Zhou et al., 2010; Zhao et al., 2011, 2013). However, the mechanism by which metabolic reprogramming contributes to drug resistance in cancer cells is currently unknown. It is thought that targeting cancer cell metabolism enzymes can improve therapy and overcome resistance to chemotherapy (Roh et al., 2016; Taniguchi et al., 2016). Targeting glycolytic pathway enzymes, such as glucose transporter (GLUT), hexokinase (HK), pyruvate kinase M2 (PKM2), and lactate dehydrogenase-A (LDHA), the first and rate-limiting of the pentose phosphate pathway (PPP) enzyme glucose-6-phosphate dehydrogenase (G6PD), and the fatty acid biosynthesis pathway enzyme fatty acid synthesis (FASN), may overcome cancer drug resistance in cancer cells (Zhou et al., 2010; Catanzaro et al., 2015).

Several lines of evidence suggest that cancer cells upregulate the oxidative PPP to support cell growth and survival. However, the role of oxidative PPP in cisplatin resistance has not been clearly described. Catanzaro et al. reported that the first and rate-limiting enzyme of the oxidative PPP, G6PD, was increased in cisplatin-resistant cells and cisplatin-resistant cells were more sensitive to death when G6PD is inhibited (Catanzaro et al., 2015). 6-phosphogluconate dehydrogenase (6PGD) is the third enzyme in the oxidative PPP, and is important for cancer cell growth (Lin et al., 2015). 6PGD has also been reported to be upregulated in many cancers (Lin et al., 2015). We previously demonstrated that 6PGD is activated by lysine acetylation, which is links the oxidative PPP, lipogenesis and tumor growth in numerous cancer cells, and thus represents a promising anti-cancer target. We developed 6PGD inhibitor Physcion, which effectively inhibits cell proliferation in numerous cancer cells with no obvious off target effect and low toxicity to normal cells (Lin et al., 2015). We also found that the combination of Physcion with anti-malarial agent dihydroartemisinin (DHA) synergistically inhibits leukemia cell growth without inducing hemolysis (Elf et al., 2016). Therefore, we reasoned that combining Physcion with chemotherapy drugs may increase the efficacy of single agent chemotherapy treatment and overcome resistance in human cancer. Although Physcion has been extensively studied in its mechanism of inhibiting diverse cancer cell proliferation in our research, it has been rarely tested in the setting of drug resistance. Therefore, the efficacy of Physcion combing with conventional chemotherapeutic agents is needs further study.

Increasing evidences suggest that microRNAs (miRNA) can control gene expression by degrading RNA or inhibiting its translation (Zhang et al., 2012; Zhu et al., 2016). The therapeutic effectiveness of cisplatin, due to the common development of cisplatin resistance, is related to dysregulation of miRNA (Chen et al., 2016; Cheng et al., 2016; Zhou et al., 2016). Fu et al. reported that miR-141 was involved in regulating cisplatin sensitivity in non-small lung cancer (NSCLC) cells via target gene PDCD4, and suppression of miR-141 might be a therapeutic strategy to overcome cisplatin resistance in lung cancer (Fu et al., 2016). MiR-1244 were significantly down-regulated in cisplatin-resistant A549/DDP cell and the rescued expression of miR-1244 reduced cell invasion and increased cell apoptosis and reverse cisplatin resistance in NSCLC cells (Li et al., 2016). Despite these advances, the correction between miRNAs and the development of ovarian and lung cancer chemoresistance remains unclear. Moreover, there are not any studies about miRNAs negatively regulating 6PGD in cisplatin resistance.

During this study, we aim to explore the metabolic mechanisms underlying cisplatin resistance in ovarian and lung cancer, which are good model to study cisplatin resistance, that is because ovarian and lung cancer patients are easy to acquire resistance to cisplatin. We showed that cisplatin-resistant cancer cells rewired cell metabolism, and that 6PGD was upregulated in cisplatin-resistant cancer cells relative to their cisplatin-sensitive counterparts. Inhibition of 6PGD by Physcion or miRNAs could reverse the resistance to the chemotherapeutic agent cisplatin. These results supported the hypothesis that cancer cells are more dependent on the oxidative PPP than normal cells and that combining inhibitors of the pentose cycle may represent a promising approach for selectively causing oxidative stress-induced cell killing in ovarian and lung cancer cells.

## Materials and Methods

### Reagents and Antibodies

Antibody against 6PGD (1:150 times dilution) (catalog number: sc-398977) and β-actin (1:500 times dilution) (catalog number: sc-47778) was from Santa Cruz Biotech; Cisplatin was purchased from Sigma–Aldrich (catalog number: P4394); Physcion was purchased from Santa Cruz Biotech (catalog number: sc-205805). The miR-206, miR-613 and miRNA Mimic Negative Control (Mimic NC, miRNA Mimic Negative Controls show the minimal homology to all known miRNAs of miRBase 18.0, and it is a crucial experimental control for miRNA “gain-of-function” studies)and miR-206 inhibitor, miR-613 inhibitor and miRNA Inhibitor Negative Control (Inhibitor NC, micrOFF^TM^ miRNA Inhibitor Negative Control is designed for minimum homology to the miRNAs being studied, and thus an indispensable control for miRNA functional studies) were purchased from RiboBio Co. Ltd. Lipofectamine RNAiMAX transfection reagent was purchased from Thermo Fisher Scientific (catalog number: 13778-150).

### Cell Culture and Establishment of Cisplatin-Resistant A549 Cells

Human ovarian surface epithelial cancer cells OV2008 and C13^∗^ were gifts from Dr. Benjamin K. Tsang (University of Ottawa, ON, Canada). OV2008 and C13^∗^ cells were cultured in RPMI 1640 medium with 10% bovine serum (FBS). Human lung adenocarcinoma epithelial cells A549 were gifts from Dr. Zhi Shi (Jinan University, Guangdong, China). A549 cells were cultured in Dulbecco Modified Eagle Medium (DMEM) with 10% FBS. Cisplatin-resistant A549 cells (A549DDP) were developed from the parental cisplatin-sensitive A549 cells, respectively, by exposure to increasing concentrations of cisplatin (cis-platinum (II) diamine dichloride [CDDP]; Sigma–Aldrich) (Seah et al., 2015). The cisplatin resistance in the established cell lines was evaluated by cell viability assays and compared to that of the parental cells. C13^∗^ stable knockdown 6PGD cell was achieved using lentiviral system (6PGD shRNA from Open Biosystems; 5′-CCGGGTGGATGATTTCATCGAGAAACTCGAGTTTCTCGATGAAATCATCCACTTTTT-3′) as previous descripted (Lin et al., 2015).

### Cell Transfection

RNA oligonucleotides were transfected using Lipofectamine RNAiMAX in a final concentration of 100 nM miR-206 or miR-613, 200 nM anti-miR-206 or anti-miR-613. RNA transfection efficiency was approximately 70–80% and the overexpression of miRNA persisted for at least 4 days.

### Cell Proliferation Assay

Cell proliferation assays were performed by seeding 5 × 10^4^ cells in 6-well plates. Relative cell proliferation was determined by cell counting at 3 days after being seeded and the percentage cell proliferation of the control. Cell growth was determined by cell numbers recorded at 0, 1, 2, 3, and 4 days after being seeded. IC50 (half maximal inhibitory concentration) was used to measure the effectiveness of an inhibitor in inhibiting a specific biological or biochemical function. Firstly, we changed the concentration of drug to logarithm, and put the relative cell proliferation and the logarithm concentration into Graphpad Prism software. Secondly, the logarithm concentration was referred as X, and the relative cell proliferation was referred as Y, then a line chart was created. Lastly, we can obtain IC50 from the Dose-response-Stimulation function in Graphpad Prism.

### Cell Viability Assay

To determine cell viability, cells were seeded in 96-well plates and treated with different concentration of DDP for 48h. Cell viability was quantified by Cell Counting Kit-8 (CCK-8; Dojindo Molecular Technologies, Inc.). Synergism effect of combinational drugs was evaluated by the Median-effect Method of Chou and Talalay. The cells were treated with a series of fixed-ratio combinations of cisplatin and Physcion. After 48h, cell viability was measured by CCK8 assay and combination index (CI) was used to determine synergy.

### Oxidative PPP Flux Assay

Oxidative PPP flux assay was used ^14^CO_2_ Release as describted previously (Lin et al., 2015). In brief, a 6-cm dish with cells was placed in a 10-cm dish with 2 sealed pinholes on the top. Cells on the 6-cm dish were treated with 2 mL of medium containing [1-^14^C] - or [6-^14^C]-glucose at 37°C for 3 h, respectively. We then injected 0.3 mL of 50% TCA through one of the holes into cells to stop the PPP flux, and at the same time injected 0.3 mL of Hyamine Hydroxide into a small cup placed on the 10-cm dish through the second hole for trapping ^14^CO_2_ release. We sealed each dish with parafilm and placed the dish at room temperature for overnight. Hyamine Hydroxide in the small cup was dissolved into 60% methanol and directly subjected to scintillation counting.

### NADPH/NADP^+^ Ratio Assay

NADPH/NADP^+^ ratio was measured by a Colorimetric Assay Kit (Sigma–Aldrich) as described previously (Lin et al., 2015). In brief, 2 × 10^6^ cells were trypsinized and washed with PBS, and lysed with 200 μL of NADP^+^ (or NADPH) extraction buffer. Lysed cells were incubated at 60°C for 5 min, the added 20 μL of assay buffer and 200 μL of the counter NADPH (or NADP^+^) extraction buffer was added to neutralize the extracts. The extracts were centrifuged at 12,000rpm for 5 mins, and the supernatants were used to check the NADPH/NADP^+^ ratio according to the manufacturer’s protocol. The absorbance at 565 nm from the reaction mixture was measured by plate reader at 0 min and 30 mins.

### Lactate Production Assay

Cellular lactate production was measured with a colorimetric-based lactate assay kit (MBL). In brief, we seeded cells in a 6 well-plate and incubated at 37°C for overnight. Media on cells was replaced with phenol red-free RPMI medium without FBS when the cells were 50% confluent. The plate was then incubated for 1 h at 37°C. After incubation, 1 mL of media from each well was assessed using the lactate assay kit. Cell numbers were counted by a microscope.

### Intracellular ATP Assay

Intracellular ATP was measured by an ATP Colorimetric/Fluorometric Assay Kit (Sigma–Aldrich) as previous described (Lin et al., 2015). In brief, 1 × 10^6^ cells were trypsinized and resuspended in ultrapure water. Luminescence was measured by a spectrofluorometer (SpectraMax Gemini; Molecular Devices) immediately after the addition of ATP enzyme mix to cell suspension.

### 6PGD Enzyme Activity Assay

6PGD enzyme activity in OV2008, C13^∗^, A549, and A549DDP cells was determined by the NADPH production rate assay as described previously (Lin et al., 2015). In brief, 6PGD enzyme activity was determined by the NADPH production rate in assay buffer (0.1 mM NADP^+^, 0.2 mM 6-phosphogluconate, 1 mM MgCl_2_, and 50 mM Tris, pH 8.1). Ten microgram of protein from cell lysate was added and the reaction was then initiated by adding NADP^+^. The increase in 340 nm absorbance (OD340) as a measure of NADPH production was obtained every 9 s for 10 min on a spectrofluorometer (SpectraMax Gemini; Molecular Device).

### Clinical Samples

The 76 ovarian cancer tissues and 23 adjacent non-tumor tissues from the cancer resection margin and 96 Non-small Cell Lung Cancer (NSCLC) tissues, and 23 adjacent non-tumor tissues were used for this study. All tissues were collected from The First Affiliated Hospital of Jinan University and Affiliated Zhongshan Hospital of Dalian University. Detail information was in the Supplementary Information.

### Ethics Statement

This study was carried out in accordance with the recommendations of Requirements of the Ethical Review System of Biomedical Research Involving Human by National Health and Family Planning Commission of China, Jinan University and Dalian University Ethics Committee with written informed consent from all subjects. All subjects gave written informed consent in accordance with the Declaration of Helsinki.

### Statistical Analysis

Statistical analyses were performed using Student’s *t*-test. Correlation between 6PGD expression and clinic pathological characteristics were evaluated by Chi-square test and Fisher’s exact tests. The survival rates after tumor removal were calculated by the Kaplan–Meier method. All data were obtained from three independent experiments performed in triplicate and were presented as the mean ± standard error. *P* < 0.05 was considered to indicate a statistically significant difference.

## Results

### Reprogrammed Metabolism in Cisplatin-Resistant Cells

**FIGURE 1:**
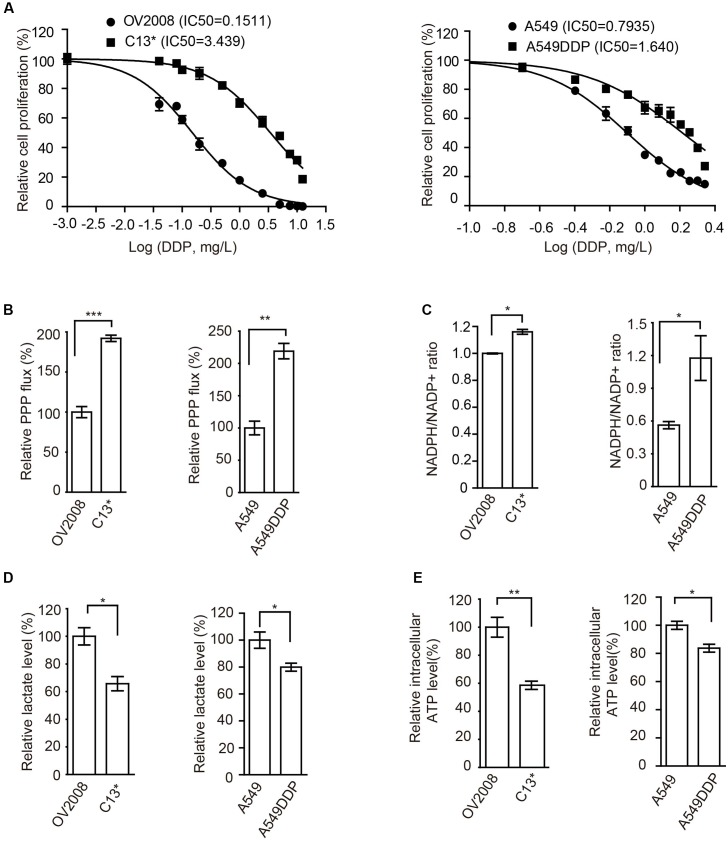
Cisplatin-resistant cells have rewired cellular metabolism. **(A)** Sensitivity of OV2008, C13^∗^, A549, and A549DDP cells upon 72 h cisplatin exposure were determined by cell counting. **(B–E)** OV2008, C13^∗^, A549, and A549DDP cells were tested for oxidative PPP flux **(B)**, NADPH/NADP^+^ ratio **(C)**, lactate production **(D)**, and intracellular ATP levels **(E)**. Error bars represent mean values ± SD from three replicates of each sample (^∗^*P <* 0.05; ^∗∗^*P* < 0.01; ^∗∗∗^*P* < 0.001).

To determine the mechanism of cisplatin resistance in cancer cells, we first compared cisplatin-resistant cancer cells C13^∗^ and A549DDP with parental OV2008 and A549 cells in terms of cisplatin sensitivity and cell metabolism. Relative cell proliferation assay results showed that A549DDP and C13^∗^ cells exhibited significantly higher resistance to cisplatin than non-DDP-resistant cells (**Figure 1A**). The IC50 of cisplatin in C13^∗^ cells was 22-fold higher than that in OV2008 cells and IC50 of cisplatin in A549DDP cells was twofold higher than that in A549 cells. This distinct effect proved to be stable, as the same results were obtained by CCK-8 assay (Supplementary Figure 1A).

We next found that, compared to cisplatin sensitive cells, cisplatin-resistant ovarian cancer C13^∗^ and lung cancer A549DDP cells showed increased oxidative PPP flux (**Figure 1B**) and NADPH/NADP^+^ ratio (**Figure 1C**), decreased glycolytic pathway lactate production (**Figure 1D**), intracellular ATP levels (**Figure 1E**), and increased ROS level (Supplementary Figure 1B). These data together suggested that cisplatin-resistant cancer cells display rewired cell metabolism.

### 6PGD Expression is Significantly Higher in Cisplatin-Resistant Cancer Cell Lines

**FIGURE 2:**
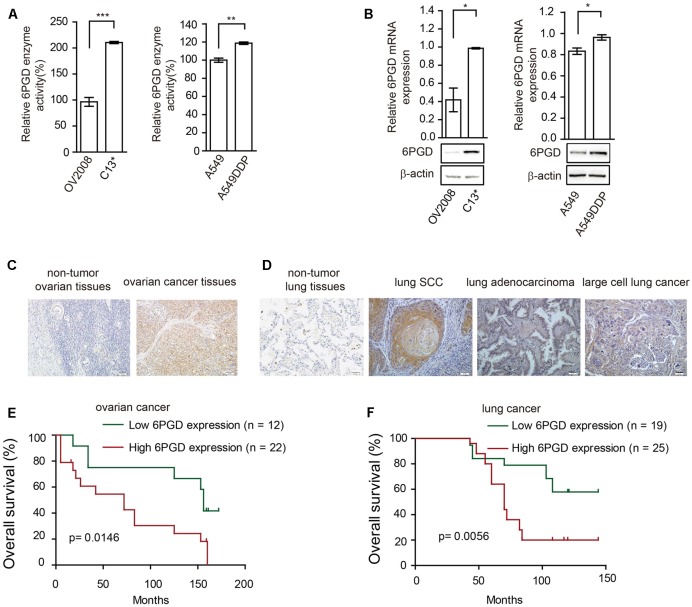
6-phosphogluconate dehydrogenase (6PGD) expression is upregulated in cisplatin-resistant cells and higher 6PGD gene expression correlates with reduced survival of cancer patients. **(A)** 6PGD enzyme activity by enzyme activity assay in OV2008, C13^∗^, A549, and A549DDP cells. **(B)** 6PGD mRNA expression levels by qRT-PCR (top) and protein levels by Western blotting (bottom) in OV2008, C13^∗^, A549, and A549DDP cells. **(C)** 6PGD protein levels were analyzed in non-tumor ovarian tissues and ovarian cancer tissues. **(D)** 6PGD protein levels were analyzed in non-tumor lung tissues and lung SCC, lung adenocarcinoma, and large cell lung cancer tissues. **(E,F)** Kaplan–Meier analysis of OS rates in 34 ovarian cancer patients **(E)** and 44 NSCLC patients **(F)** relative to 6PGD protein expression. Error bars represent mean values ± SD from three replicates of each sample (^∗^*P <* 0.05; ^∗∗^*P* < 0.01; ^∗∗∗^*P* < 0.001).

We next asked whether 6PGD is involved in reprogramming metabolism in cisplatin-resistant cells. Firstly, we compared the expression and enzyme activity between cisplatin-resistant and cisplatin-sensitive cells. Our results showed that 6PGD enzyme activity was upregulated in cisplatin-resistant C13^∗^ and A549DDP cells (**Figure 2A**). 6PGD expression level was also increased in cisplatin-resistant cells (**Figure 2B** and Supplementary Figure 1C). Taken together, these results suggested that cisplatin-resistant cells might upregulate 6PGD expression as a cisplatin resistant mechanism.

### Higher 6PGD Gene Expression Correlates with Reduced Survival of Ovarian and Lung Cancer Patients

To validate the clinical relevance of our findings, we analyzed the relationship between 6PGD expression levels and clinical outcomes of ovarian cancer and lung cancer patients. Immunohistochemistry results showed that the 6PGD protein was mostly expressed in the cytoplasm of ovarian cancer cells as well as lung squamous cell carcinoma (SCC), lung adenocarcinoma and large cell lung cancer cells (**Figures 2C,D**). The expression of 6PGD was positive in 53 (69.7%) and strongly positive in 36 (47.4%) of the 76 ovarian patients, which was significantly higher than in adjacent non-tumor ovarian tissues (30.4%, 7/23; 13.0%, 3/23) (Supplementary Table 1). Additionally, the positive rate of the 6PGD protein expression was 88.5% (85/96) and strongly positive rate of 6PGD expression was 65.3% (61/96) in NSCLC tissues, which was significantly higher than that in adjacent non-tumor tissues (21.7%, 5/23; 4.3%, 1/23) (Supplementary Table 4).

We then analyzed the correlation between 6PGD protein and the clinicopathological parameters. High 6PGD protein expression tends to be strongly correlated with clinical stage and lymph node metastasis in ovarian cancer. For clinical stage, the strongly positive rate of 6PGD protein was significantly higher in stage III–IV ovarian cancer tissues (63.2%, 24/38) than in stage I–II (31.6%, 12/38). The strongly positive rate of 6PGD protein was higher in ovarian cancers with lymph node metastasis (73.7%, 14/19) compared with those with no metastasis (38.6%, 22/57). However, no statistical differences were found between 6PGD expression and patient age, tumor size, and M classification (Supplementary Tables 2, 3).

Moreover, the positive rate of 6PGD protein was related to clinical stage and lymph node metastasis in lung cancer. The strongly positive rate of 6PGD protein was significantly higher in high grade NSCLC than in cases with low histological grade. Similarly, we found that the strongly positive rate of 6PGD protein was significantly higher in stages II-IV (72.9%, 51/70) than those in stages I (38.5%, 10/26). Additionally, it was also higher in adenocarcinoma and squamous carcinoma tissues (69.4%, 25/36; 70.0%, 28/40) than in other cases (40.0%, 8/20). However, there were no significant correlations between 6PGD expression and lymph node metastasis, gender, and patient’s age in NSCLC (Supplementary Tables 5, 6).

To further authenticate the significance of 6PGD expression in ovarian cancer and NSCLC progression, we evaluated the relationship between 6PGD positive expression and overall survival (OS) rate in 34 ovarian cancer cases and 44 NSCLC cases using the Kaplan-Meier method, and found that patients with 6PGD high expression had drastically reduced OS than those with 6PGD low expression (**Figures 2E,F**).

### Low Expression of miR-206 and miR-613 in Cisplatin Resistant Cells Correlates with Increased 6PGD in Cisplatin Resistant Cells

**FIGURE 3:**
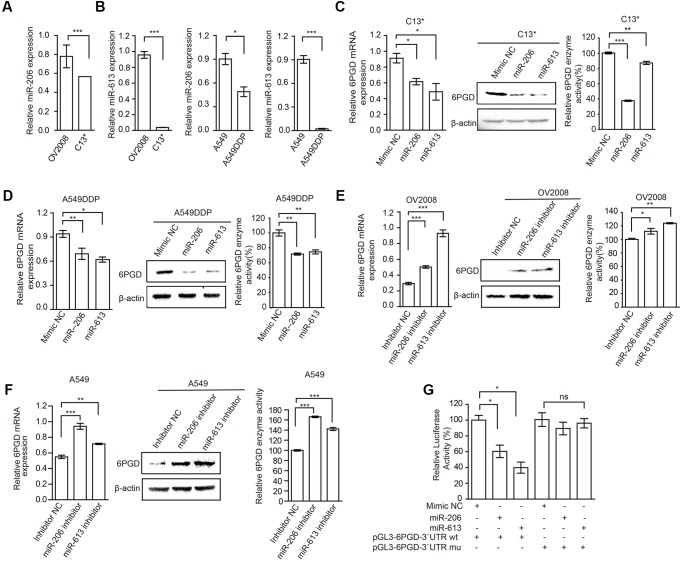
MiR-206 and miR-613 directly inhibit the expression of 6PGD through its 3′UTR. **(A,B)** qRT-PCR analysis of miR-206 and miR-613 expression in OV2008 and C13^∗^ cells **(A)** and A549 and A549DDP cells **(B)**. **(C,D)** The effects of miRNAs on 6PGD mRNA, protein expression and enzyme activity, were determined at 48 h after transfected with 100 nM Mimic Negative control (Mimic NC) and 100 nM miR-206 and miR-613 in C13^∗^ **(C)** and A549DDP **(D)** cells, respectively. **(E,F)** The effects of miRNA inhibitors on 6PGD mRNA, protein expression and enzyme activity, were determined at 48 h after transfected with Inhibitor negative control (Inhibitor NC) and miR-206 inhibitor and miR-613 inhibitor in OV2008 cells **(E)** and A549 cells **(F)**, respectively. **(G)** 293T cells were co-transfected with Renilla luciferase plasmid and a firefly luciferase reporter plasmid containing either wild-type or mutant 6PGD 3′UTR (indicated as pGL3-6PGD-3′UTR-wt and pGL3-6PGD-3′UTR-mu) with either control or miR-206 or miR-613. Luciferase activity was conducted at 24 h after transfection. Error bars represent mean values ± SD from three replicates of each sample (^∗^*P <* 0.05; ^∗∗^*P* < 0.01; ^∗∗∗^*P* < 0.001).

To explore the mechanisms underlying increased 6PGD expression in cisplatin-resistant cells, we predicted target miRNAs of 6PGD in human microRNA.org-Targets and Expression^1^. Among them, we are particularly interested in miR-206 and miR-613, because of their high mirSVR score (Supplementary Figure 1D). To further confirmed and validated this finding, we compared the expression of miR-206 and miR-613 in cisplatin-resistant cells with their counterparts. The data showed that miR-206 and miR-613 were downregulated in cisplatin-resistant cells (**Figures 3A,B**), when compared to their counterparts. To test whether miR-206 and miR-613 expression affected endogenous 6PGD expression, we transfected miR-206 and miR-613 or Mimic NC into cisplatin resistant-cells, and observed decreased 6PGD mRNA and protein levels and enzyme activity (**Figures 3C,D**). Consistent with these results, silencing of miR-206 and miR-613 in cisplatin sensitive cells led to an increase in 6PGD mRNA and protein levels, as well as enzyme activity (**Figures 3E,F**). As shown in Supplementary Figure 1D, it was predicted that a single miR-206 and miR-613 binding site on the 3′UTR of 6PGD mRNA at nucleotides 42–48, which was highly conserved among different species. To validate whether 6PGD was a direct target of miR-206 and miR-613, we constructed pGL3-6PGD-3′UTR-wt and pGL3-6PGD-3′UTR-mu plasmids and performed a 3′UTR reporter-binding assay (Supplementary Figure 1D lower). The overexpression of miR-206 or miR-613 significantly reduced the pGL3-6PGD-3′UTR-wt luciferase activity but not that of pGL3-6PGD-3′UTR-mu in 293T cells (**Figure 3G**).

**FIGURE 4:**
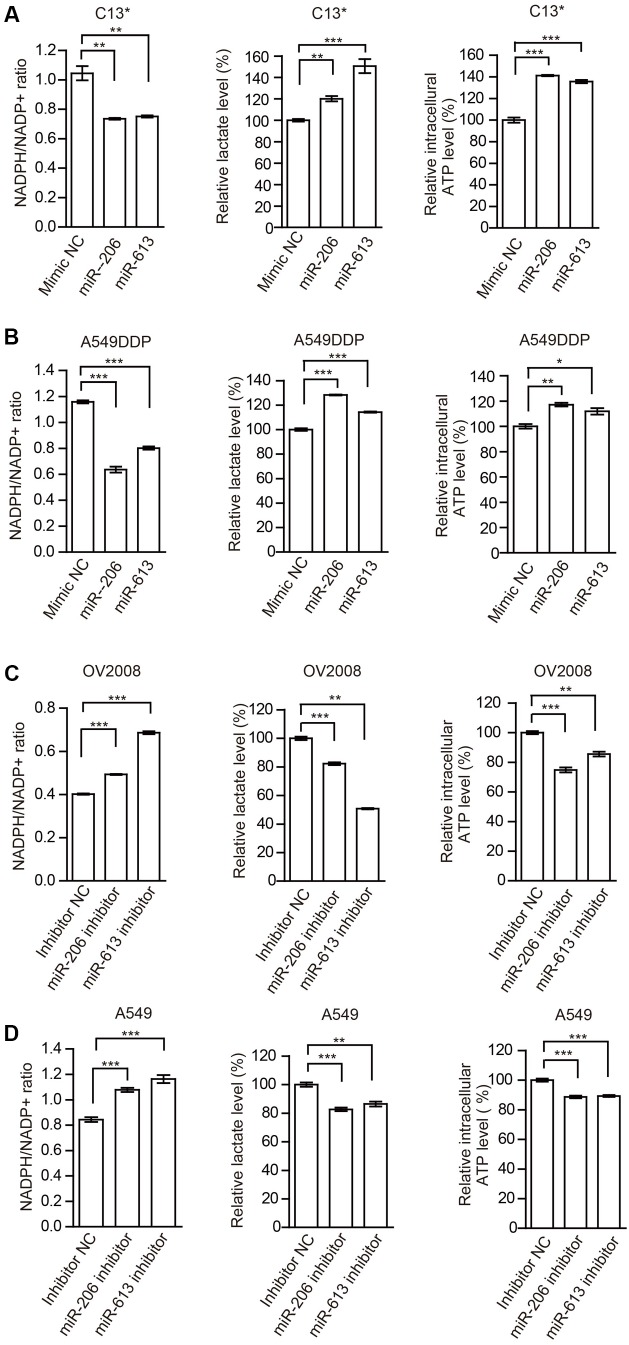
miR-206 and miR-613 have rewired cellular metabolism by targeting 6PGD. **(A,B)** C13^∗^**(A)** and A549DDP **(B)** cell lines were transfected with Mimic NC, miR-206 and miR-613, respectively. The effects of miRNAs on NADPH/NADP^+^ ratio, lactate production and intracellular ATP levels were determined. **(C,D)** OV2008 **(C)** and A549 **(D)** cell lines were transfected with Inhibitor NC and miR-206 inhibitor and miR-613 inhibitor, respectively. The effects of miRNA inhibitors on NADPH/NADP^+^ ratio, lactate production and intracellular ATP levels were determined. Error bars represent mean values ± SD from three replicates of each sample (^∗^*P <* 0.05; ^∗∗^*P* < 0.01; ^∗∗∗^*P* < 0.001).

In addition, we examined the effects of miR-206 and miR-613 on metabolic reprogramming, and found that decreased NADPH/NADP^+^ ratio, increased glycolytic pathway lactate production and intracellular ATP levels in cisplatin-resistant cells that were transfected with miR-206 and miR-613 (**Figures 4A,B**). At same time, we observed that increased NADPH/NADP^+^ ratio, decreased lactate production, and decreased intracellular ATP levels in cisplatin sensitive cells which transfected with miR-206 and miR-613 inhibitors (**Figures 4C,D**). These results suggest that miR-206 and miR-613 play an important roles in metabolic reprogramming. Taken together, these data indicated that the cisplatin-resistant cancer cell attenuated miR-206 and miR-613 to increase 6PGD expression, leading to metabolic reprogramming.

### Inhibition of 6PGD Sensitizes Cisplatin-Resistant Cells

To investigate whether 6PGD inhibition could delay the emergence of cisplatin resistance in ovarian and lung cancer cells, we next examined whether Physcion (6PGD inhibitor) (Supplementary Figure 2A) could reverse the cisplatin resistance in ovarian and lung cancer cells and enhance its anti-tumor effects. First, we examined cell viability of cisplatin-resistant C13^∗^ and A549DDP cells with increased concentrations of Physcion and found that Physcion inhibited cell viability with IC50 values of approximately 24.21 and 55.23 μM in C13^∗^ and A549 DDP, respectively (Supplementary Figure 2B). Then we found that Physcion treatment resulted in decreased cell proliferation and 6PGD enzyme activity of OV2008, C13^∗^, A549 and A549DDP cells in a dose dependent manner (Supplementary Figures 2C–F).

**FIGURE 5:**
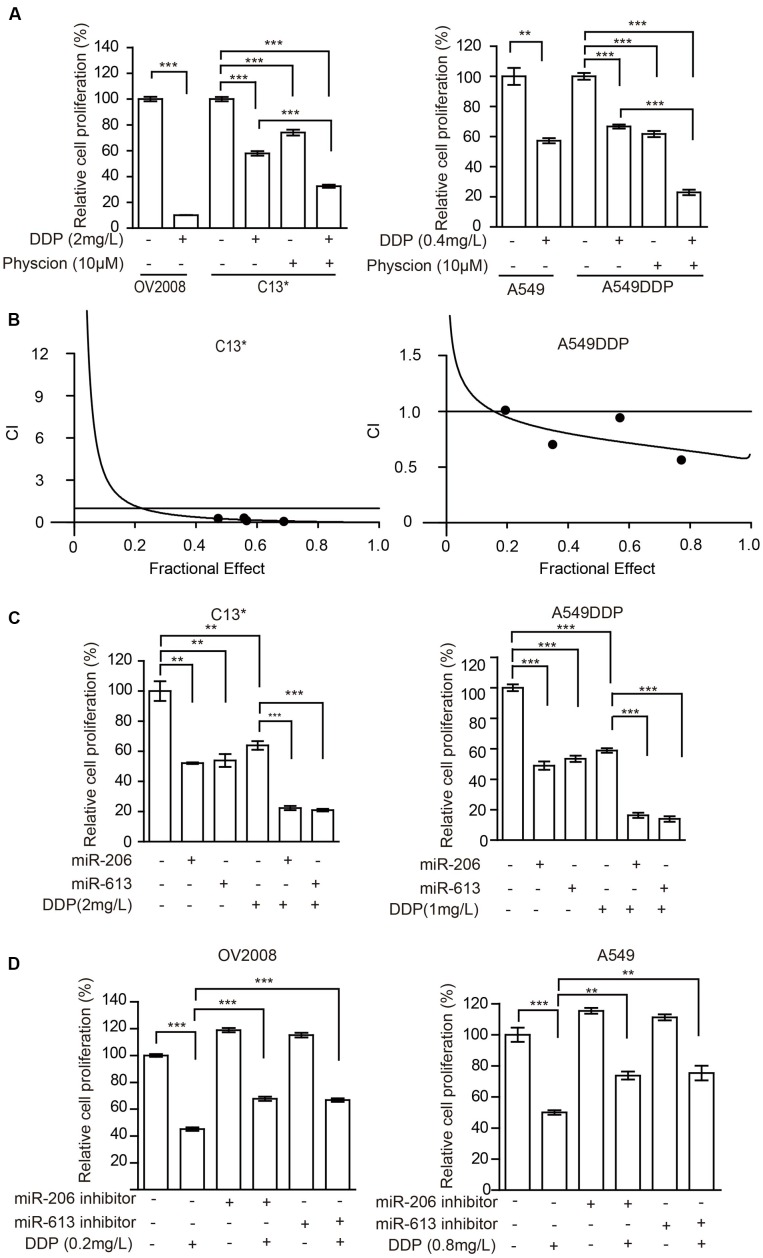
Inhibition of 6PGD can reverse cisplatin resistance in cancer cell lines. **(A)** OV2008/C13^∗^ or A549/A549DDP cells were treated with Physcion or DDP alone or in combination and cell proliferation was determined by cell counting. **(B)** C13^∗^ and A549DDP cells were treated with a serial constant-ratio combining Physcion and DDP for 48 h. The cell viability-based synergistic effect of Physcion and DDP was analyzed according to approach described by Chou and Talalay. The combination index (CI) smaller than 1 indicates a synergetic effect. **(C)** C13^∗^ and A549DDP cells treated with miRNA-206/ miRNA-613 or DDP alone or in combination and cell proliferation was determined by cell counting. **(D)** OV2008 and A549cells treated with miRNA-206/miRNA-613 inhibitor or DDP alone or in combination and cell proliferation was determined by cell counting. Error bars represent mean values ± SD from three replicates of each sample (^∗^*P <* 0.05; ^∗∗^*P* < 0.01; ^∗∗∗^*P* < 0.001).

The cancer cells were then incubated with a combination of Physcion and cisplatin at concentrations below the IC50. Treatment of C13^∗^ cells with cisplatin showed 41% inhibition of cell proliferation, whereas cisplatin almost completely inhibited cell proliferation in OV2008 cells. Interestingly, pretreatment of C13^∗^ cells with Physcion significantly reversed cisplatin resistance (**Figure 5A** left). We made similar observations in A549DDP cells (**Figure 5A** right). We further tested the synergistic effect of cisplatin and Physcion by the Median-effect Method described by Chou and Talalay. Synergism (CI < 1) was observed between the two agents (**Figure 5B**).

We then tested whether miR-206 or miR-613 could sensitize the resistant-cells to cisplatin. We found that pretreatment of C13^∗^ and A549DDP cells with miR-206 or miR-613 mimic significantly reversed cisplatin resistance (**Figure 5C**) and pretreatment of OV2008 and A549 cells with miR-206 or miR-613 inhibitors significantly render resistance to cisplatin (**Figure 5D**). Taken together, these results suggest that inhibition of 6PGD results in increased sensitivity to cisplatin in ovarian and lung cancer cells.

## Discussion

Targeting of cancer cell metabolism combined with conventional chemotherapeutic agents is particularly appealing because most cancer cells take up more glucose to produce diverse precursors of biomass, which include lipids, nucleotides, and amino acids, that support rapid cell proliferation (Vander Heiden et al., 2010; Vander Heiden, 2011). Unfortunately, mounting evidence supports the idea that deregulated cancer cell metabolism, including increased aerobic glycolysis, fatty acid biosynthesis and glutamine metabolism, can sustain drug resistance (Liu et al., 2008). Cisplatin is currently one of the most commonly used chemotherapeutic drugs for treating ovarian and lung cancer, through regulating diverse pathways, including apoptosis, cell proliferation, cell cycle arrest, DNA repair, the TCA cycle and glycolysis (Pabla and Dong, 2008; Xu et al., 2008; Alborzinia et al., 2011). However, an understanding of how cisplatin affects metabolic pathways is still in its infancy.

In this study, we analyzed the metabolic reprogramming of cisplatin-resistant cells metabolic reprogramming, including PPP, glycolysis, and oxidative phosphorylation. Cisplatin-resistant cells exhibited increased PPP flux, NADPH/NADP^+^ ratio, and ROS, and decreased lactate production and intracellular ATP. Catanzaro et al reported that cisplatin-resistant cells relied on the PPP to overcome cisplatin cytotoxicity, and inhibition of the first and rate-limiting enzyme of the PPP, G6PD, reverse cisplatin resistance (Catanzaro et al., 2015). Consistent with this view we found that the expression and enzymatic activity of 6PGD, the third enzyme of the PPP, were also increased in resistant cells compared to their sensitive counterparts. Additionally, patients with higher 6PGD expression display reduced progression free survival compared to patients with low 6PGD expression levels.

The deregulations of miRNAs played vital roles in the modulation of diverse cellular processes, including chemoresistance. To investigate the mechanism of 6PGD expression upregulation in cisplatin-resistant cells, we analyzed the target miRNA of 6PGD and found that miR-206 and miR-613 were downregulated in cisplatin-resistant cells. We further characterized 6PGD as a functional target of miR-206 and miR-613 by luciferase reporter gene assays, qRT-PCR and western blot analysis, respectively. Furthermore, miR-206 and miR-613 could reprogram cell metabolism through targeting 6PGD. Therefore, we could conclude that miR-206 and miR-613 played pivotal roles in regulating 6PGD expression and reprogramming metabolism in cisplatin-resistant cells.

Recently, several studies have highlighted the importance of combination treatment with cisplatin plus inhibitors targeting metabolic enzymes to improve resistance to chemotherapy (Zhou et al., 2010; Catanzaro et al., 2015). In the present study, we found that cisplatin resistance in ovarian and lung cancer cells can be reverse by targeting PPP enzyme 6PGD, which has a crucial role in regulating cancer cell metabolism. The combined treatment with the 6PGD inhibitor Physcion and cisplatin showed a selective synergistic effect on cisplatin-resistant cells, suggesting that upregulation of 6PGD activity could be a promising targetable mechanism underlying cisplatin resistance in cancer cells. Moreover, when we treated cisplatin-resistant cancer cells with miRNA-206/miRNA-613 and cisplatin, we found that this presented a predominate effect on cisplatin-resistant cells. Taken together, our data clearly indicated that targeting a PPP enzyme offers a novel synthetic lethality approach for overcoming cisplatin resistance.

**FIGURE 6:**
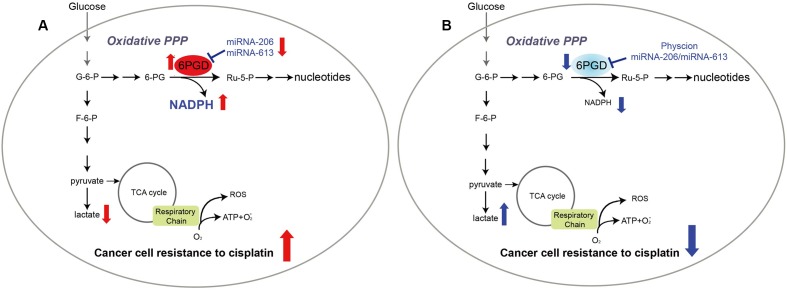
6-phosphogluconate dehydrogenase offers superiority for cisplatin resistance. Proposed working model.**(A)** In cisplatin-resistant cancer cells, miRNA-206 and miRNA-613 are commonly downregulated in cisplatin-resistant cells, leading to reduced expression levels of 6PGD. And reprogramming cell metabolism, including NADPH/NADP+ ratio, lactate production and intracellular ATP levels, to fulfill cancer cells resistant to cisplatin treatment. **(B)** Attenuation of 6PGD by miRNAs or small molecule inhibitor Physcion results in decreased 6PGD expression or enzyme activity, leading to cancer cell sensitive to cisplatin treatment.

In summary, our results showed that profound metabolic changes in cisplatin-resistant cancer cells. 6PGD is commonly upregulated in cisplatin-resistant cells due to decrease miR-206 and miR-613, and leads to fulfill cancer cells resistance to cisplatin treatment (**Figure 6A**). Attenuation of 6PGD by miRNAs or small molecule inhibitor Physcion resulted in decreased 6PGD expression or enzyme activity, leading to cancer cell sensitivity to cisplatin treatment (**Figure 6B**). Our study demonstrated that the combination of cisplatin treatment with targeting 6PGD could remarkably improve the effects of cisplatin and can help to overcome cancer resistance to cisplatin treatment.

## Author Contributions

WZ, QF, and JL: Perform and analyze all the experiments. YG, LG, RL, MX, GY, and ZY drafted the work for important intellectual content. SZ, SL, and CS: Writing of the manuscript and designed the study.

## Conflict of Interest Statement

The authors declare that the research was conducted in the absence of any commercial or financial relationships that could be construed as a potential conflict of interest.

## Footnotes

1 http://www.microrna.org/microrna/home.do
