# Supplementary material for: Inhibition of 6-phosphogluconate Dehydrogenase Reverses Cisplatin Resistance in Ovarian and Lung Cancer
Source: Front Pharmacol. 2017 Jun 30;8:421. doi: 10.3389/fphar.2017.00421 (PMC5491617; doi:10.3389/fphar.2017.00421)
Supplement: Supplementary file 1 [file Table_1.PDF]

Supplementary Table 1. Expression of 6PGD protein in ovarian cancer

Diagnosis	No. Of case	6PGD				Positive cases rate (%)	Strong positive cases rate (%)
		-	+	++	+++		
Ovarian cancer	76	23	17	14	22	69.7%**	47.4%**
Adjacent normal ovarian	23	16	4	3	0	30.4%	13.0%

Positive rate: percentage of positive cases with +, ++, and +++ staining score.

Strongly positive rate (high-level expression): percentage of positive cases with ++ and +++ staining score.

** p<0.01 compared with adjacent normal ovarian.
