# Supplementary material for: Inhibition of 6-phosphogluconate Dehydrogenase Reverses Cisplatin Resistance in Ovarian and Lung Cancer
Source: Front Pharmacol. 2017 Jun 30;8:421. doi: 10.3389/fphar.2017.00421 (PMC5491617; doi:10.3389/fphar.2017.00421)
Supplement: Supplementary file 2 [file Table_2.PDF]

Supplementary Table 2. Relationship between 6PGD protein overexpression and the clinicopathological features of ovarian cancer

**Variables**	**No. Of case (*n*)**	**6PGD Strong positive rate (%)**	χ^2	***P* value**
**Age (years)**				
≥50	44	18 (40.9%)	1.749	0.186
<50	32	18 (56.3%)		
**Tumor size**				
≤ 4 cm	35	15 (42.9%)	0.530	0.467
＞4 cm	41	21 (51.2%)		
**M classification**				
M0	50	22 (44.4%)	0.665	0.415
M1	26	14 (53.8%)		
**Lymph node status**				
N0	57	22 (38.6%)	7.037	0.008**
N+	19	14 (73.7%)		
**Clinical stage**				
Ⅰ+Ⅱ	38	12(31.6%)	7.600	0.006**
Ⅲ+Ⅳ	38	24 (63.2%)		

* $p<0.05$ and ** $p<0.01$
