# Supplementary material for: Inhibition of 6-phosphogluconate Dehydrogenase Reverses Cisplatin Resistance in Ovarian and Lung Cancer
Source: Front Pharmacol. 2017 Jun 30;8:421. doi: 10.3389/fphar.2017.00421 (PMC5491617; doi:10.3389/fphar.2017.00421)
Supplement: Supplementary file 3 [file Table_3.PDF]

Supplementary Table 3. Cox regression model analysis of the clinicopathological features in 34 patients with ovarian cancer

**Characteristics**	**B**	**SE**	**Wald**	**HR**	**95%CI**		***P* value**
					Lower	Upper	
**Univariate**							
Age	0.590	0.405	2.124	1.803	0.816	3.984	0.145
Tumor size	0.260	0.358	0.526	1.297	0.643	2.616	0.468
M	0.030	0.345	0.007	1.030	0.524	2.026	0.932
LN	1.257	0.436	8.310	3.514	1.495	8.257	0.004**
Clinical stage	0.807	0.366	4.848	2.240	1.093	4.593	0.028*
6PGD	1.130	0.422	7.167	3.097	1.354	7.086	0.007**
**Multivariate**							
LN	0.852	0.476	3.202	2.344	0.922	5.959	0.074
Clinical stage	0.852	0.428	3.961	2.344	1.013	5.423	0.047*
6PGD	1.299	0.466	7.780	3.667	1.472	9.136	0.005*

B: coefficient; SE: standard error; Wald: Waldstatistic; HR: hazard ratio; CI: confidence interval

* p<0.05 and ** p<0.01

#
