# Supplementary material for: Inhibition of 6-phosphogluconate Dehydrogenase Reverses Cisplatin Resistance in Ovarian and Lung Cancer
Source: Front Pharmacol. 2017 Jun 30;8:421. doi: 10.3389/fphar.2017.00421 (PMC5491617; doi:10.3389/fphar.2017.00421)
Supplement: Supplementary file 4 [file Table_4.PDF]

Supplementary Table 4. Expression of 6PGD protein in Lung cancer

Diagnosis	No. of case	6PGD				Positive cases rate (%)	Strong positive cases rate (%)
		-	+	++	+++		
Lung cancer	96	11	24	19	42	88.5%**	63.5%**
Adjacent normal lung	23	18	4	1	0	21.7%	4.3%

Positive rate: percentage of positive cases with +, ++, and +++ staining score.

Strongly positive rate (high-level expression): percentage of positive cases with ++ and +++ staining score.

**** p<0.01 compared with adjacent normal lung.**
