# Supplementary material for: Inhibition of 6-phosphogluconate Dehydrogenase Reverses Cisplatin Resistance in Ovarian and Lung Cancer
Source: Front Pharmacol. 2017 Jun 30;8:421. doi: 10.3389/fphar.2017.00421 (PMC5491617; doi:10.3389/fphar.2017.00421)
Supplement: Supplementary file 5 [file Table_5.PDF]

Supplementary Table 5. Relationship between 6PGD protein overexpression and the clinicopathological features of lung cancer

Variables	No. of case (*n*)	6PGD strong positive rate (%)	χ^2	*P* value
**Age (years)**				
>50	66	43 (65.2%)	0.236	0.627
≤50	30	18 (60.0%)		
**Gender**				
Male	64	43 (65.2%)	0.236	0.627
Female	32	18 (60.0%)		
**Histological**				
Adenocarcinoma	36	25 (69.4%)	6.046	0.049*
Squamous cell carcinoma	40	28 (70.0%)		
Others	20	8 (40%)		
**Lymph node status**				
N0	30	15 (40.0%)	3.454	0.063
NX	66	46 (74.2%)		
**Histological grade**				
Grade-1	19	4 (21.1%)	18.894	0.000**
Grade-2	40	31 (77.5%)		
Grade-3	37	26 (70.3%)		
**TNM stage**				
I	26	10 (38.5%)	9.682	0.002**
II~IV	70	51 (72.9%)		

*** p<0.05 and ** p<0.01**
