# Supplementary material for: Inhibition of 6-phosphogluconate Dehydrogenase Reverses Cisplatin Resistance in Ovarian and Lung Cancer
Source: Front Pharmacol. 2017 Jun 30;8:421. doi: 10.3389/fphar.2017.00421 (PMC5491617; doi:10.3389/fphar.2017.00421)
Supplement: Supplementary file 6 [file Table_6.PDF]

Supplementary Table 6. Cox regression model analysis of the clinicopathological features in 44 patients with lung cancer

Characteristics	B	SE	Wald	HR	95%CI		*P* value
					Lower	Upper	
**Univariate**							
Age	0.291	0.329	0.780	1.337	0.702	2.549	0.377
Gender	0.152	0.315	0.234	1.164	0.628	2.158	0.628
Histological	0.464	0.321	2.085	1.590	0.847	2.986	0.149
Grade	0.041	0.226	0.033	1.042	0..669	1.623	0.856
LN	0.636	0.332	3.675	1.888	0.986	3.618	0.055
TNM	0.713	0.342	4.351	2.040	1.044	3.987	0.037*
6PGD	0.742	0.319	5.426	2.101	1.125	3.923	0.020*
**Multivariate**							
TNM	0.645	0.342	3.547	1.501	0.974	3.730	0.060
6PGD	0.688	0.321	4.601	2.240	1.061	3.734	0.032*

B: coefficient; SE: standard error; Wald: Waldstatistic; HR: hazardratio; CI: confidenceinterval

* p<0.05 and ** p<0.01
