# Supplementary material for: Inhibition of 6-phosphogluconate Dehydrogenase Reverses Cisplatin Resistance in Ovarian and Lung Cancer
Source: Front Pharmacol. 2017 Jun 30;8:421. doi: 10.3389/fphar.2017.00421 (PMC5491617; doi:10.3389/fphar.2017.00421)
Supplement: Supplementary file 7 [file Presentation_1.PDF]

Inhibition of 6-phosphogluconate dehydrogenase reverses cisplatin resistance in ovarian and lung cancer cell

Running title: Targeting 6PGD reverse cisplatin resistance

Wujian Zheng[1,†], Qi Feng[1,†], Jiao Liu[1,†], Yanke Guo[1], Lvfen Gao[2], Ruiman Li[2], Meng Xu[3], Guizhen Yan[4], Zhinan Yin[1], Shuai Zhang[5,*], Shuangping Liu[6,*] and Changliang Shan[1,*]

[1] The First Affiliated Hospital, Biomedical Translational Research Institute, Jinan University, Guangzhou, Guangdong 510632, China.

[2]Department of Gynecology and Obstetrics, The First Affiliated Hospital, Jinan University, Guangzhou, Guangdong 510632, China. [3]Department of Oncology, The First Affiliated Hospital, Jinan University, Guangzhou, Guangdong 510632, China.

[4]Lixia District People's Hospital, Jinan, Shandong 250014, China.

[5] Department of Biochemistry and Molecular Biology, Medical College of Jinan University, Guangzhou, Guangdong 510632, China.

[6]Department of Pathology, Medical School, Dalian University, Dalian, Liaoning 116622, China.

[*]Correspondence: Shuai Zhang, shuaizhang@jnu.edu.cn; Shuangping Liu, liushuangping@dlu.edu.cn; Changliang Shan, changliangshan@jnu.edu.cn.

[†] These authors contributed equally to this study.

Supplementary Materials and methods

Intracellular Reactive Oxygen Species (ROS) Production

The amount of intracellular ROS was measured by detecting

dichlorodihydrofluorescein as previous descripted (Lin et al., 2015).

Real-time quantitative reverse transcription-PCR

Total cellular RNA was extracted using the Eastep® Super RNA Extract reagent Kit (Promega). cDNA was generated from purified RNA using PrimeScipt^{TM} RT reagent Kit (Takara) according to the manufacturer's instructions. 6-phosphogluconate dehydrogenase (6PGD) cDNA was amplified by PCR using the following primers: 5′-CATCGAGAAATTGAGACGGTG -3′ (forward) and 5′-CCTTGGAAGATGGTCTT AT -3′ (reverse). For β-actin (an endogenous control), the following primers were used: 5′-ACGTGGACATCCGCAAAG-3′ (forward) and 5′-GACTCGTCATACTCCTGCTTG -3′ (reverse). Real-time quantitative reverse transcription-PCR (qRT-PCR) was performed using SYBR Green (Biotool) on a Bio-rad CFX96 Realtime PCR System (Bio-rad). Relative target mRNA levels were normalized to β-actin expression.

Luciferase reporter gene assay

Luciferase reporter assay was performed using the Dual-Luciferase Reporter Assay System (Promega) according to the manufacturer`s instructions. When the cells density reach 70% confluence after seeded in 24-well plates. For 6PGD 3'UTR luciferase reporter gene assay, we co-transfected with 100 nM Negative Control (NC) or miR-206 or miR-613, pGL3-6PGD -3'UTR-wt or pGL3-6PGD 3'UTR-mut and Renilla luciferase plasmids using PEI (Sigma-Aldrich). Cells were lysed and assayed for luciferase activity 48 hours after transfection. 100 μl of protein extracts were analyzed in a luminometer.

Western blot analysis

Cells were lysed with lysis buffer (1.5M NaCl, 1M HEPES[pH=7.0], 1%NP40, 0.1M$Na_4P_2O_7$, 0.1M NaF, 0.1M Na_3VO_4, protease inhibitor) on ice 30 min and then centrifuged at 12,000 rpm for 15 min at 4°C. Protein samples were separated by 12%SDS-PAGE and transferred onto PVDF membranes (Millipore). The membranes were blocked with 5 % non-fat milk for 2 hours and then incubated overnight at 4 °C with the primary antibody and 1 hour at room temperature with secondary antibody. Signals were detected using Luminol substrate solution.

Clinical samples

All tissues were routinely fixed in 10% buffered formalin and embedded in paraffin blocks. The study protocol was approved by the institutional review board of Dalian University Medical College. The pathological parameters, including gender, age, tumor size, clinical stage, differentiation, nodal metastasis and survival data were carefully reviewed. All cases were confirmed by pathological examination. The tumor, node, and metastasis (TNM) staging was assessed according to the staging system established by the American Joint Committee on Cancer (AJCC).

The ovarian cancer patients include 76 females, with a median age of 50 years (range, 42-75 years). A total of 76 patients, 44 cases were 50 years old or over and 32 cases were below 50 years old. Of the 76 ovarian cancer patients, 38 cases were stages I while 38 cases were stages II-IV. In addition, 50 cases have no distant metastasis and 26 cases have distant metastasis. Additionally, 19 cases have lymph node (LN) metastasis, and 57 cases have no LN metastasis (Supplementary Table 2).

The patients with NSCLC include 64 males and 32 females, and ranging from 45 to 71 years with a mean age of 57 years. A total of 96 patients, 66 cases were 50 years old or over and 30 cases were below 50 years old. Of the 96 NSCLC, 26 cases were stages I while 70 cases were stages II-IV, and for the histological grade, 19 cases were defined as grade-1, 40 cases were grade-2 and 37 cases were grade-3. In addition, 36 cases were defined as adenocarcinoma, while 40 cases as squamous cell carcinoma and 20 cases as others. Additionally, 66 cases have lymph node (LN) metastasis, and 30 cases have no LN metastasis (Supplementary Table 5). None of the patients received radio-chemotherapy before surgery. The 34 ovarian cancer patients and 44 patients with NSCLC had been followed up for 172 months and 144 months or until death.

Immunohistochemistry (IHC) for 6PGD protein in paraffin-embedded tissues

IHC analysis was performed using the DAKO LSAB kit (DAKO A/S, Glostrup, Denmark). Briefly, to eliminate endogenous peroxidase activity, 4 μm thick tissue sections were deparaffinized, rehydrated and incubated with 3% H_2O_2 in methanol for 15 min at RT. The antigen was retrieved at 95°C for 20 min by placing the slides in 0.01 M sodium citrate buffer (pH 6.0). The slides were then incubated with 6PGD antibody (1:500, BD Biosciences Pharmingen) at 4 °C overnight. After incubation with biotinylated secondary antibody at RT for 30 min, the slides were incubated with streptavidin-peroxidase complex at RT for 30 min. IHC staining was developed by using 3,3′-diaminobenzidine, and Mayer’s hematoxylin was used for counterstaining. In addition, the positive tissue sections were processed with omitting of the primary

antibody as negative controls.

Evaluation of IHC staining

All specimens were examined by two investigators (S Liu & C Shan) who did not possess knowledge of the clinical data. In case of discrepancies, a final score was established by reassessment on a double-headed microscope. Briefly, the IHC staining for 6PGD was semi-quantitatively scored as '-' (negative, no or less than 5% positive cells), '+' (5-50% positive cells), and '++' (more than 50% positive cells, considered as strongly positive). Only the cytoplasmic expression pattern was considered as positive staining.

Supplementary Figures Legends

Supplementary Figure 1. (A) C13*/A549DDP and OV2008/A549 were treated with different concentrations of DDP and cell viability were determined by CCK8 assay. (B) C13*/A549DDP and OV2008/A549 cells were tested for ROS levels. (C) 6PGD mRNA expression levels by RT-PCR in C13* (A549DDP) cells compared to OV2008 (A549) cells. (D) miR-206 and miR-613 binding site is predicted at 42-48nt of the 3`UTR of mRNA. Mutant was generated at the 3`UTR seed region as indicated. The 3`UTR fragment containing wild type or mutant of the miR-206 or 613binding sequence was cloned into the downstream of the luciferase reporter gene. Error bars represent mean values±SD from three replicates of each sample (**P*<0.05; ***P*<0.01; *** *P*<0.001).

Supplementary Figure 2. Inhibition of 6PGD can reverse cisplatin resistance in cancer cell lines. (A) Structure of Physcion. (B) Cell viability of C13*and

A549DDP cells in the presence of increasing concentrations of Physcion were determined by CCK8. (C-F) Cell proliferation rates (left) and 6PGD enzyme activity (right) of C13* cells (C), OV2008 cells (D), A549 cells (E) and A549DDP cells (F) in the presence of increasing concentrations of Physcion were determined by cell counting and enzyme activity assay. Error bars represent mean values ± SD from three replicates of each sample (*P<0.05; **P<0.01; *** P<0.001).
