# Supplementary figures and images for: Inhibition of 6-phosphogluconate Dehydrogenase Reverses Cisplatin Resistance in Ovarian and Lung Cancer
Source: Front Pharmacol. 2017 Jun 30;8:421. doi: 10.3389/fphar.2017.00421 (PMC5491617; doi:10.3389/fphar.2017.00421)

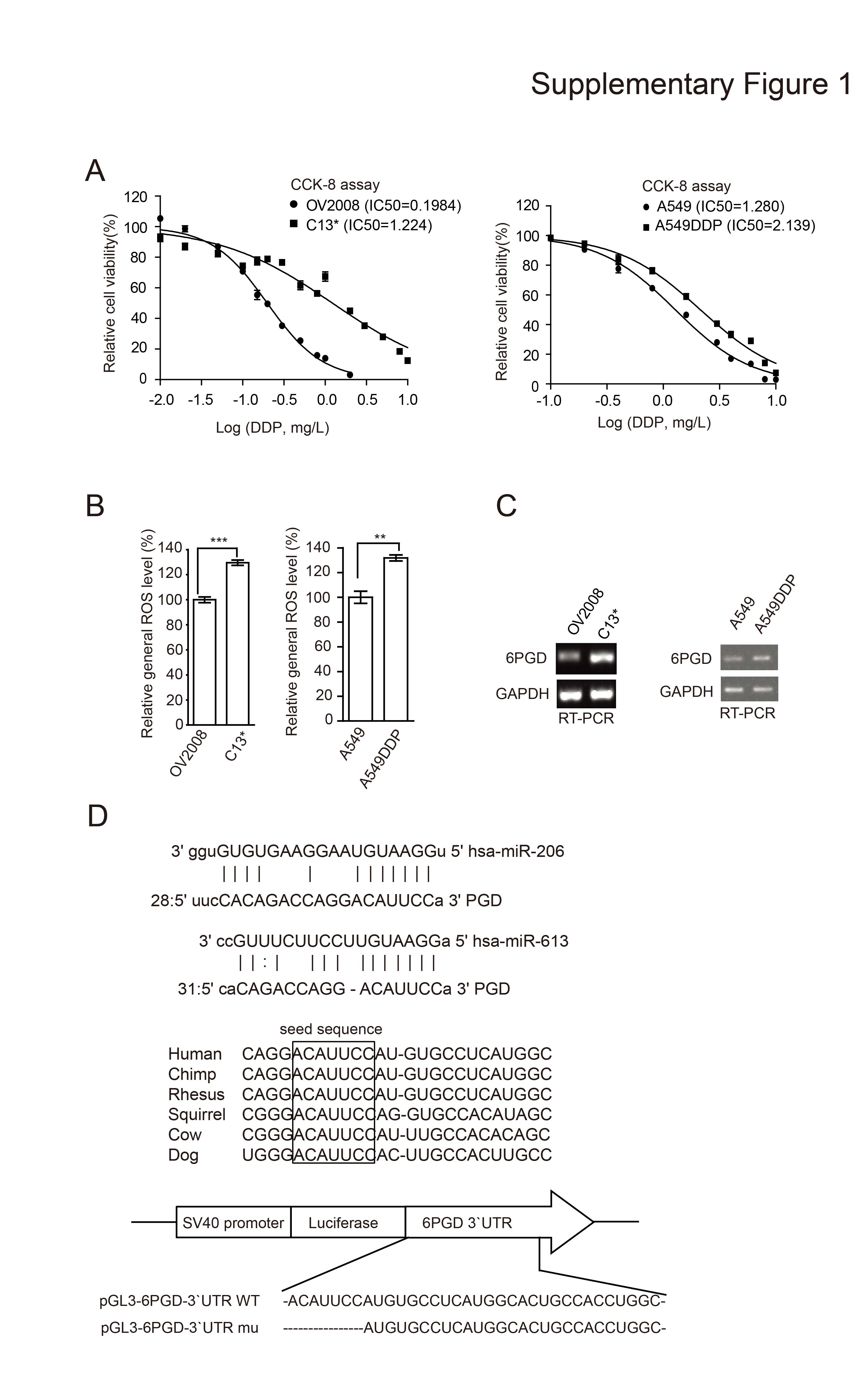

Supplement: Supplementary file 8 [file Image_1.JPEG]

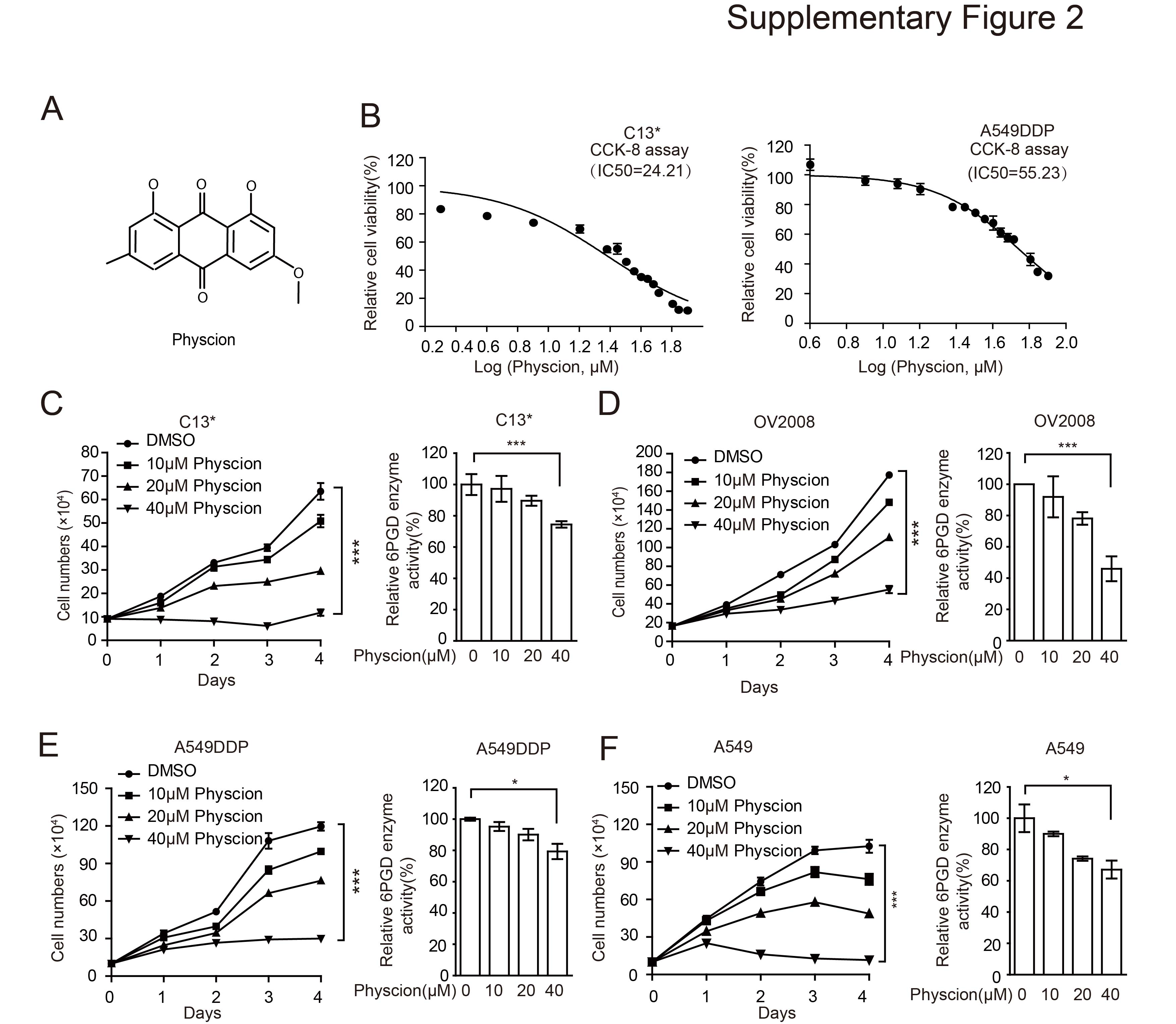

Supplement: Supplementary file 9 [file Image_2.JPEG]
